# miR-199a/b-3p inhibits HCC cell proliferation and invasion through a novel compensatory signaling pathway DJ-1\Ras\PI3K/AKT

**DOI:** 10.1038/s41598-023-48760-8

**Published:** 2024-01-02

**Authors:** Li-Na Ma, Li-Na Wu, Shuai wei Liu, Xu Zhang, Xia Luo, Shah Nawaz, Zi min Ma, Xiang-Chun Ding

**Affiliations:** 1 Department of Infectious Diseases, General Hospital of Ningxia Medical University, Ningxia Sinasheng Biotechnology Co. LTD, Yinchuan, 750004 Ningxia China; 2 Ningxia Medical University, Yinchuan, Ningxia China; 3 Ningxia Sinasheng Biotechnology Co. LTD, Yinchuan, Ningxia China

**Keywords:** Cancer, Cell biology, Computational biology and bioinformatics, Medical research, Oncology

## Abstract

Several studies have reported the effects of DJ-1 gene and miR-199a/b-3p on HCC development. However, whether miR-199a/b-3p regulates HCC progression through a novel compensatory signaling pathway involving DJ-1, Ras, and PI3K/AKT remains unknown. We used (TCGA, HPA, miRWalk and Target scan) databases, cancer and para-tissue HCC patients, dual-luciferase reporter gene analysis, proteomic imprinting, qPCR, cell proliferation, scratch, transport, and flow cytometry to detect the molecular mechanism of DJ-1 and miR-199a/b-3p co-expression in HCC cell lines. Bioinformatics analysis showed that DJ-1 was highly expressed in HCC ((*P* < 0.001) were closely associated with tumor stage (T), portal vein vascular invasion, OS, DSS, and PFI (*P* < 0.05); miR-199a/b-3p was lowly expressed in HCC (*P* < 0.001), which was the upstream regulator of DJ-1. Spearman coefficient r = −0.113, *P* = 0.031; Dual luciferase gene report verified the negative targeting relationship between them P&lt; 0.001; Western blotting demonstrated that miR-199a/b-3p could inhibit the protein expression of DJ-1, Ras and AKT(*P* < 0.05); The results of CCK8, cell scratch, Transwell migration and flow cytometry showed that OE + DJ-1 increased the proliferation, migration and invasion ability of HepG2 cells, and decreased the apoptosis process, and the differences were statistically significant (*P* < 0.05), while miR-199a/b-3p had the opposite effect (*P* < 0.05).

## Introduction

Primary Liver cancer (PLC) is the sixth most common malignant solid tumor in the world and the third leading cause of cancer-related death^1^. In China, approximately 320,000 people die each year from PLC, in which hepatocellular carcinoma (HCC) accounting for about 75%^2,3^. Despite developing in HCC diagnosis and treatment recently, the 5-year overall survival rate remains low, approximately in 11.7–14.1%^1–3^. Since the activation of proto-oncogenes and/or inactivation of suppressor genes of tumor play significant roles in regulating HCC proliferation, apoptosis, invasion and metastasis^4–7^,so identifying key targets and molecular mechanisms is critical for the success of antitumor drug development.

DJ-1 protein, encoded by the mitogen-dependent oncogene PARK7^8^, is a highly conserved protein expressed in various tissue cells. It plays important roles in oxidative stress clearance, mitochondrial function maintenance, and apoptosis regulation^9–11^. Based on research, it has been found that a lack of DJ-1 protein can increase the risk of developing neurodegenerative diseases, such as Parkinson's disease and Alzheimer diseases^12,13^. DJ-1 has become an important protein in the study of neurological diseases, oxidative stress, and cell survival, as well as a potential therapeutic target^8–13^. Additionally, DJ-1 has been found to act as a "chaperone" and can work with oncogenes like c-MYC or H-Ras to promote the proliferation, apoptosis, invasion, and metastasis of various types of cancers^14–26^. It's worth noting that the expression level of DJ-1 in HCC has been linked to survival time, according to studies ^27–32^, and is inversely correlated with the tumor suppressor gene PTEN and that it can regulate HCC proliferation, apoptosis, invasion, and metastasis by directly affecting MAPK/AKT or indirectly acting on the PI3K/AKT signaling pathway.The components of these signaling cascades have been targeted for therapeutic intervention. However, recent studies have revealed the presence of a compensation loop for DJ-1 regulation, which is strongly associated with the development of acquired resistance to tyrosine protein kinase anti-HCC drugs^33^.

miR-199a/b-3p, which is a tumor suppressor gene, can inhibit the proliferation of gastric cancer^34^, HCC^35–38^, breast cancer^40^, colorectal cancer^41^, and prostate cancer^42^ by regulating the PAK4/MEK/ERK signaling pathway. Additionally, it has been found miR-199a/b-3p can inhibit HCC growth, apoptosis, invasion and angiogenesis via multiple targets, such as PDCD4, CD44, CD151, VEGFA AEGFR1/2, HGF and MMP2^35–38^ and can affect the chemical sensitivity of hepatoma cells to doxorubicin via mTOR and c-Met^39^. What's more interesting to us is that DJ-1 might be a promising target gene for miR-199a/b-3p and intersect with the Ras and PI3K/AKT signaling pathways through analysis of miRWalk, TargetScan, and TGCA databases.

However, it remains unknown whether miR-199a/b-3p serves as an upstream regulatory factor for DJ-1 expression and whether it regulates a new compensatory pathway in the HCC process throughthrough a novel compensatory signaling pathway involving miR-199a/b-3p\DJ-1\Ras\PI3K/AKT. In this study, we investigated the clinical correlation between the DJ-1 gene and miR-199a/b-3p expression in hepatocellular carcinoma (HCC) using multiple bioinformation databases (miRWalk, TargetScan, TCGA), clinical tissue specimens, and HepG2 cells. We also explored the co-expression of miR-199a/b-3p and DJ-1 in HepG2 cells and their impact on biological function, as well as the expression of key proteins and genes in the Ras protein and PI3k/AKT signaling pathway. To confirm the interaction between miR-199a/b-3p and the DJ-1 gene, we employed real-time quantitative PCR (RT-qPCR), luciferase reporter gene detection experiments, and Western blotting.

## Materials and methods

### Bioinformation databases

By using TCGA, HPA, miRWalk, and TargetScan databases, the differential expression, pathological data and molecular correlation of DJ-1 gene in HCC and normal human liver tissue samples, as well as upstream miRNAs screening and signal pathway prediction of DJ-1 gene were analyzed.

### Immunohistochemical detection of DJ-1 protein in hepatocellular carcinoma and paracancer tissues

5 pairs of liver cancer and adjacent tissue samples were collected from patients with hepatocellular carcinoma after surgery in the Department of Hepatobiliary Surgery of Ningxia Medical University General Hospital. The tissue specimens were fixed in formalin solution and embedded with paraffin wax. Each sample was then sectioned into 4um thick slices, which were subjected to standard dewaxing and hydration procedures. Antigen retrieval was performed using target search solution (Dako, CA), followed by blocking of endogenous peroxidase activity with 0.3% hydrogen peroxide for 15 min. The slices were then blocked with goat serum, avidinin solution, and biotin solution, and incubated overnight at 4 °C with rabbit anti-human DJ-1 primary antibody (dilution 1:1000). High-sensitivity streptavidin-HRP conjugate detection and biotinylated goat anti-rabbit secondary antibody (Vector Laboratories CA) were used for detection. The slices were then incubated in Tris–HCl buffer with 1% H2O2 for 30 min, followed by DAB color development and counterstaining with hematoxylin QS (Vector Laboratories, CA). Finally, professional pathologists analyzed and interpreted the findings.

In accordance with the Declaration of Helsinki on Human Research, with the approval of the Ethics Committee of Ningxia Medical University General Hospital and the informed consent of patients and their guardians.In this paper, patient information is concealed.

### Cell culture

LO2, 293 T cells, and HepG2 were obtained from Nanjing Symbiotic Biotechnology and the Chinese Academy of Sciences of Shanghai Cell Bank and cultured in DMEM containing 10% FBS (containing 4.0 mM L-Glutamine, 100 U•mL-1 penicillin, and 100 ug•mL-1 streptomycin), 37 oC, 5% CO2 saturated humidity incubator.

### Western blot detection

Collect the cultured cells and extract total cell protein using RIPA lysate. Quantify the protein concentration using BCA assay. Prepare a 10% SDS-PAGE gel for protein electrophoresis and transfer the protein onto a membrane. Block the membrane with 5% skim milk at room temperature, and then incubate with primary antibody (dilution ratio 1:1000) overnight at 4 °C. The next day, incubate the membrane with HRP-labeled secondary antibody (dilution ratio 1:5000), and wash with PBST three times for 10 min each. Finally, visualize the protein bands using SuperSignalWest Pico Plus and image with a BIO RAD gel imager. Analyze the grayscale values of the protein bands using Image Lab software. This method serves as an internal reference.

### Real-time PCR detection

The experimental procedure involved the extraction of total RNA following the instructions of the TB Green Premix Ex TaqII (Tli RNaseh Plus) (Code No-RR820A) kit. The RNA was then reverse transcribed into cDNA using quantitative PCR, and real-time PCR (RT-qPCR) was performed using the LightCycler 480 system. The Ct value was obtained from the PCR instrument software, and the relative expression of mRNA was calculated using the 2-△△Ct method (△Ct = Ct target gene—△Ct internal control, △△Ct = △Ct experimental group—△Ct control group). Statistical analysis was carried out, and all experiments were repeated three times.

### Diluciferase reporter gene detection

The diluciferase reporter detected the targeted relationship between miR-199a/b-3p and DJ-1 gene, and DJ-1 was recombinant with the enzyme-digested vector after PCR amplification, and the carrier overlapping sequence was synthesized by ELK biotechnology (DJ-1 primer design as follows):

**Table:** 

DJ-1	5’-3’Primer sequences
DJ-1 F	CGTGTAAAGATCCGGTACCGCAGCGAACTGCGACGATCAC
DJ-1 R	CTCCTCGAGGATATCGGATCCCCTCATTGGTATTTTTTAAT

Culture tool cells (293 T) were grouped according to the plasmid vector constructed at target sites that could bind to the DJ-1 gene predicted by miR-199a-3p: A(NC), B(pGL6-DJ1-3'UTR-WT + pRL-TK), C (mimics NC + pGL6-DJ1-3'UTR-WT + pRL-TK), D (miR-199a-3p mimics + pGL6-DJ1-3'UTR-WT + pRL-TK), E(pGL6-DJ1-3`UTR-Mut2 + pRL-TK), F(mimics NC + pGL6-DJ1-3`UTR-Mut2 + pRL-TK), G(miR-199a-3p mimics + pGL6-DJ1-3`UTR-Mut2 + pRL-TK) ; Mimic (or miR-NC) & (or) pGL6-DJ1(/pGL6)& pRL-TK were transfected with 50 μl Opti-MEM low-serum medium, and then the RLU value obtained by firefly luciferase assay was divided by the RLU value obtained by Renilla luciferase assay with Renilla luciferase as an internal reference. The miR-199a-3p and U6 primer sequences were designed and synthesized by Ribo Technology Co., Ltd. (Guangzhou). miR-199a-3p sequence: ATTGGTTACACGUCUGAUGACG.

### Detection of the effect of miR-199a-3p and DJ-1 gene intervention on HepG2 cell function

miR-199a-3p mimics, miR-199a-3p inhibitor and overexpressed DJ-1 gene lentiviral plasmid vectors were grouped into: D2(HepG2), E2(miR-199a-3p mimics), F2 (OE-DJ-1), G2(miR-199a-3p mimics + OE-DJ-1), H2(miR-199a-3p inhibitor), I2 (miR-199a-3p inhibitor + OE-DJ-1), HepG2 cell transfection culture.

### CCK-8 cell proliferation assay

HepG2 cells were cultured in a complete medium 37 °C, 5% CO2 incubator, after trypsinization, prepared into a cell suspension with a concentration of 1 × 105 cells/mL, seeded in a 96-well plate according to 1 × 104 cells/well, add 100 μL per well, and continue to culture to the cell adherence. Replace each group of medium with 100 μL of serum-free basal medium containing 1% BSA, and after 12 h of starvation treatment, replace each group of medium with 100 μL of medium containing a certain concentration of drug corresponding to each cell sample, and set the corresponding blank zeroing wells (that is, only the corresponding medium has no cells). Add 10 μL of CCK-8 solution to all wells, incubate in an incubator for 1–4 h, determine the absorbance value at 450 nm using a microplate reader, use solvent-treated cells as the control group (D2), blank zeroing wells as blank, calculate the survival rate of drugs to cells according to the formula, and repeat the experiment (*n* = 3). Inhibition rate % = [(control group-blank)-(experimental group-blank)]/(control group-blank)*100%.

### Transwell and cell scratch experiments

HepG2 cell migration and invasion ability detection using Transwell chamber, culture HepG2 cells, place 200L of cell suspension in transwell chamber, add 500L of complete medium containing 10% FBS to the 24-well plate, place the chamber in the plate, culture in the incubator for 48 h, wash off the medium with PBS, fix paraformaldehyde for 20 min, wash 2 times with PBS, stain 0.5% crystal violet for 10 min, wash off crystal violet, Under an inverted microscope, photograph the non-cellular seeding side. The experiment was carried out three times. Scratch experimental cell culture with the previous use of marker pen at the bottom of the 6-well plate evenly draw parallel straight lines, the cells are made into 2.5*105 / mL suspension, 2 mL is added to each well of the 6-well plate, the cells are crossed out with the tip along the direction perpendicular to the horizontal line of the marker's stroke, the scratched cells are removed, the corresponding serum-free medium is added, and the picture is taken under an inverted microscope to take pictures of the intersection of the horizontal line of the marker stroke and the horizontal line of the cell drawn by the tip of the gun, and this time point is recorded as 0 h. Continue to culture for 24 h, and take pictures again around the intersection point taken at 0 h, and this time point is recorded as 24 h. The experiment was repeated 3 times.

### Cell flow cytometry to detect apoptosis

Culture HepG2 cells, digest the cells with EDTA-free pancrepsin, centrifuge the cells at 4 °C for 5 min, wash the cells with pre-chilled PBS 2 times, resuspend the cell pellet with 300uL of 1 × Binding Buffer, add 5uL Annexin V-FITC, mix and incubate in the dark for 10 min, add 5 uL PI, mix well and incubate in the dark for 5 min, machine detection within 1 h, FITC excitation 494 nm emission 520 nm, PI Excitation of 493 nm emits 636 nm. The experiment is repeated 3 times.

### Expression of DJ-1, Ras protein and AKT signaling protein molecules in PI3K/AKT signaling pathway

Same method 1.7 for cell culture and grouping,Then, protein imprinting experiments were used to detect the expression of AKT signaling protein molecules in DJ-1, Ras protein and PI3K/AKT signaling pathway in each group of cells.

### Statistical methods

SPSS26.0 was used to analyze the experimental results; data conforming to the positive Pacific distribution were expressed inX- ± S, the independent sample t-test was used to compare the two groups, and the Mann-WhitneyU test was used for the non-positive Pacific distribution sample, and *P* < 0.05 was considered statistically significant.

### Ethic approval and consent to participate

This study was approved by the Ethics Committee of Ningxia Medical University General Hospital (approval number: KYLL 2021–85) .Patients were consented by an informed consent process that was reviewed by the Ethics Committee of Ningxia Medical University General Hospital and certify that the study was performed in accordance with the ethical standards as laid down in the 1964 Declaration of Helsinki.

## Results

### Correlation analysis of DJ-1 expression with HCC

**Figure 1:**
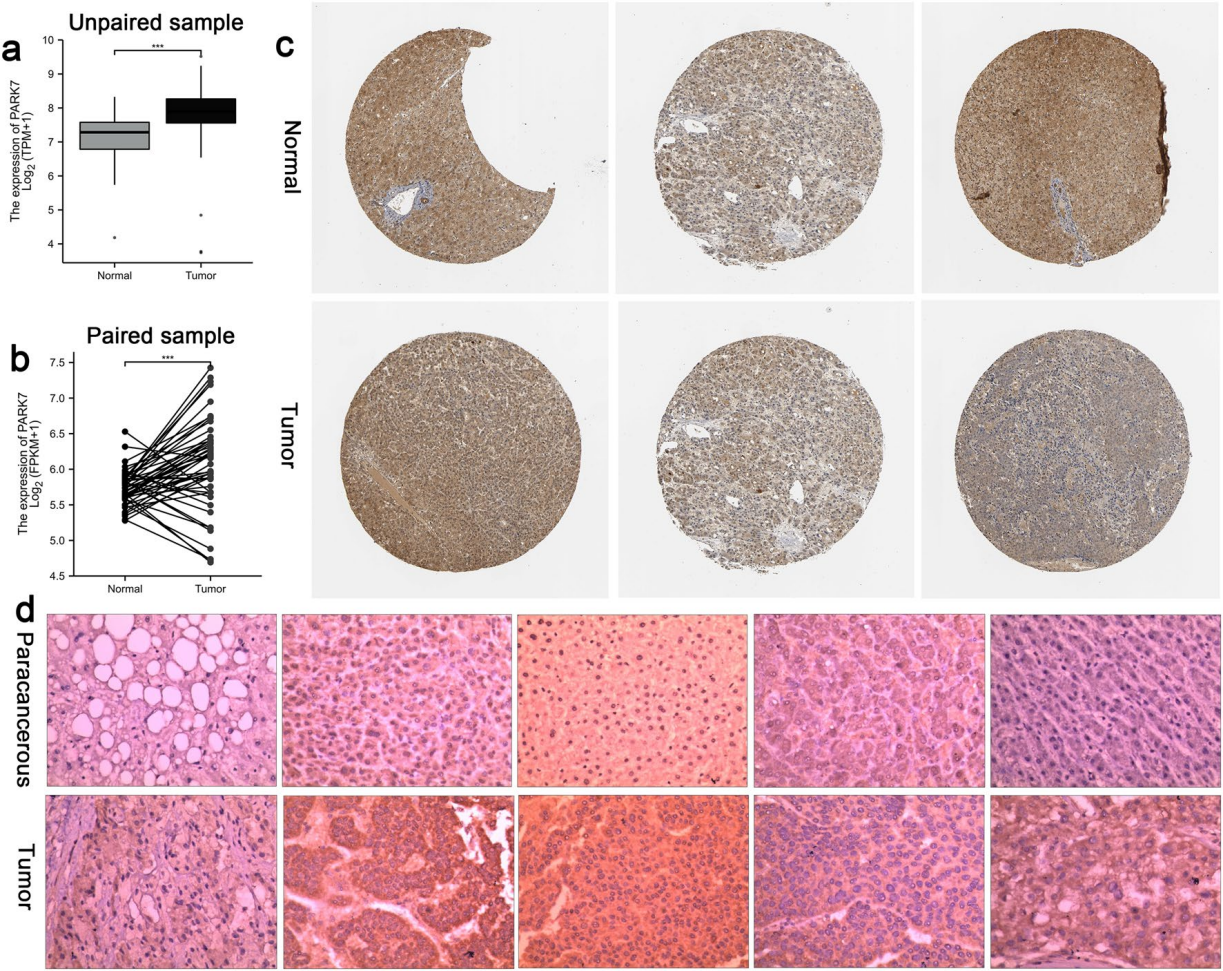
DJ-1 expression was significantly higher in cancer tissues of HCC patients compared to adjacent tissues and normal liver tissue, and the difference was statistically significant, *P* < 0.001 (**a**,**b**) DJ-1 gene expression in HCC in TCGA database. (**c**) DJ-1 IHC results of HCC tissue and normal liver tissue in the HPA database,Patient information(Normal Patient No: 1720,1899,1846,Tumor Patient No:879,1163,983,Antibody:HPA004190); d:DJ-1 IHC results in three pairs of paired clinical HCC samples.

**Figure 2:**
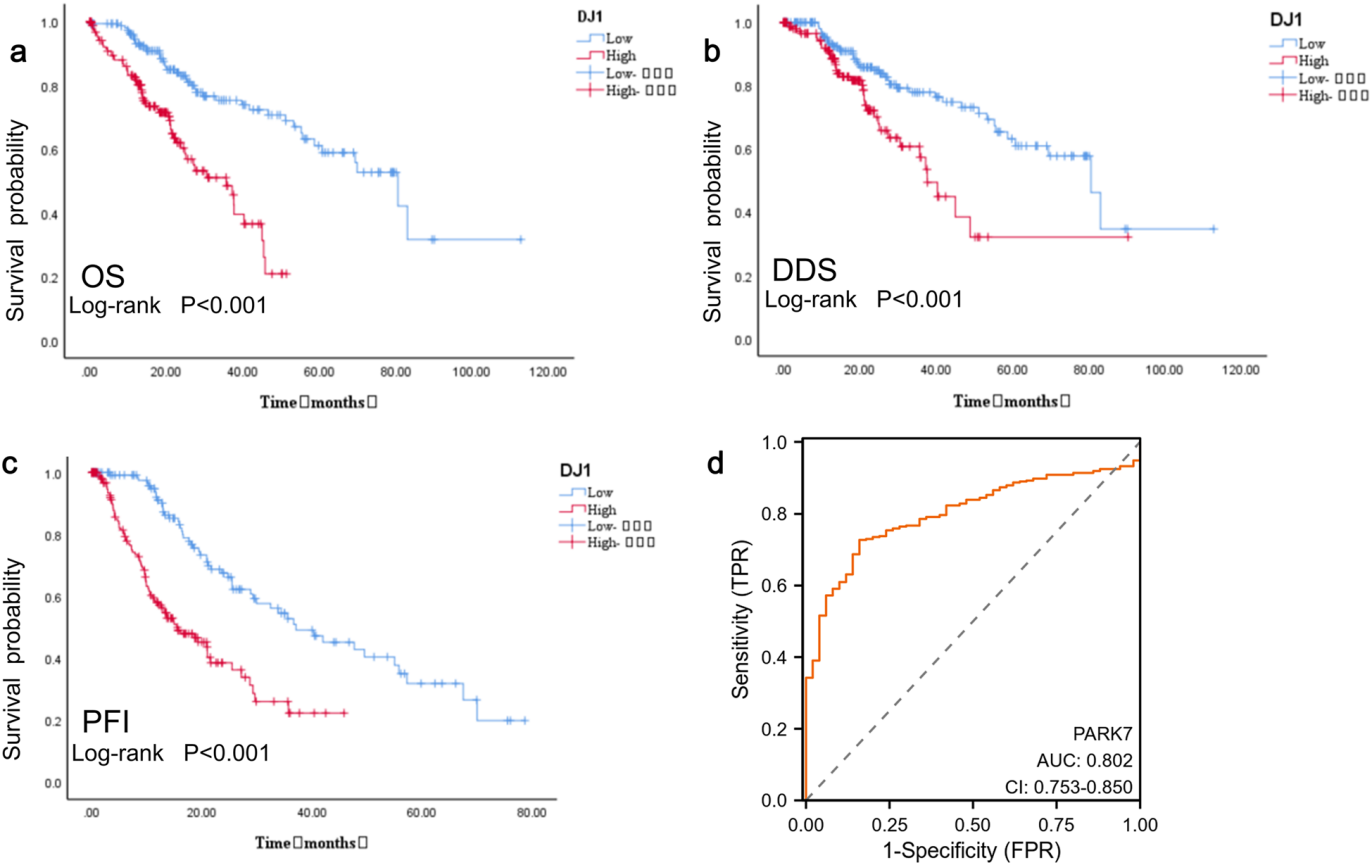
The correlation between DJ-1 expression and OS,DSS,PFI and value for clinical diagnosis in HCC.

The expression level of the DJ-1(PARK7) in HCC were analyzed by using the TCGA-LIHC( including normal liver tissue 50 cases, paracancerous 50 cases, cancer tissue 374 cases), HPA database and immunohistochemical testing. Results showed that DJ-1 expression was significantly higher in cancer tissues of HCC patients compared to adjacent tissues and normal liver tissue, and the difference was statistically significant, *P* < 0.001 ( as shown in Fig. 1a, b ,c and d) . The expression levels of DJ-1 mRNA in TNM stage III and IV HCC tissues ,with vascular invasion ,and pathological grading of 3 and 4 were higher than those in TNM stage I and II tissues(*P* < 0.01), without vascular invasion, and grading of 1 and 2(*P* < 0.05).

(as shown in Table 1). The KM curve and ROC curve analysis showed that the expression level of DJ-1in HCC was significantly associated with overall survival (OS), disease-specific survival (DSS), and tumor progression-free interval (PFI) (Fig. 2a-c). The diagnostic ROC curve demonstrated that DJ-1 had predictive value for clinical diagnosis, with an area under the curve of 79.6%, sensitivity of 84%, and specificity of 71.7% (Fig. 2d).

**Table 1:** The correlation between DJ-1 expression and HCC TNM staging, pathological grading, and vascular invasion.

Characters		n	DJ-1mRNA expression level	t	***P***
TNM staging	T1 + T2	249	72.669 ± 25.142	−2.920	**0.004**
	T3 + T4	90	82.142 ± 29.935		
Pathological grading	StageI + II	229	71.761 ± 24.769	−3.479	**0.001**
	StageIII + IV	86	83.343 ± 30.105		
Vascular invasion	Yes	198	73.461 ± 27.673	−2.687	**0.008**
	No	109	82.988 ± 33.137		

Significant values are in bold.

## Prediction, validation, and molecular correlation analysis of miRNAs

**Figure 3:**
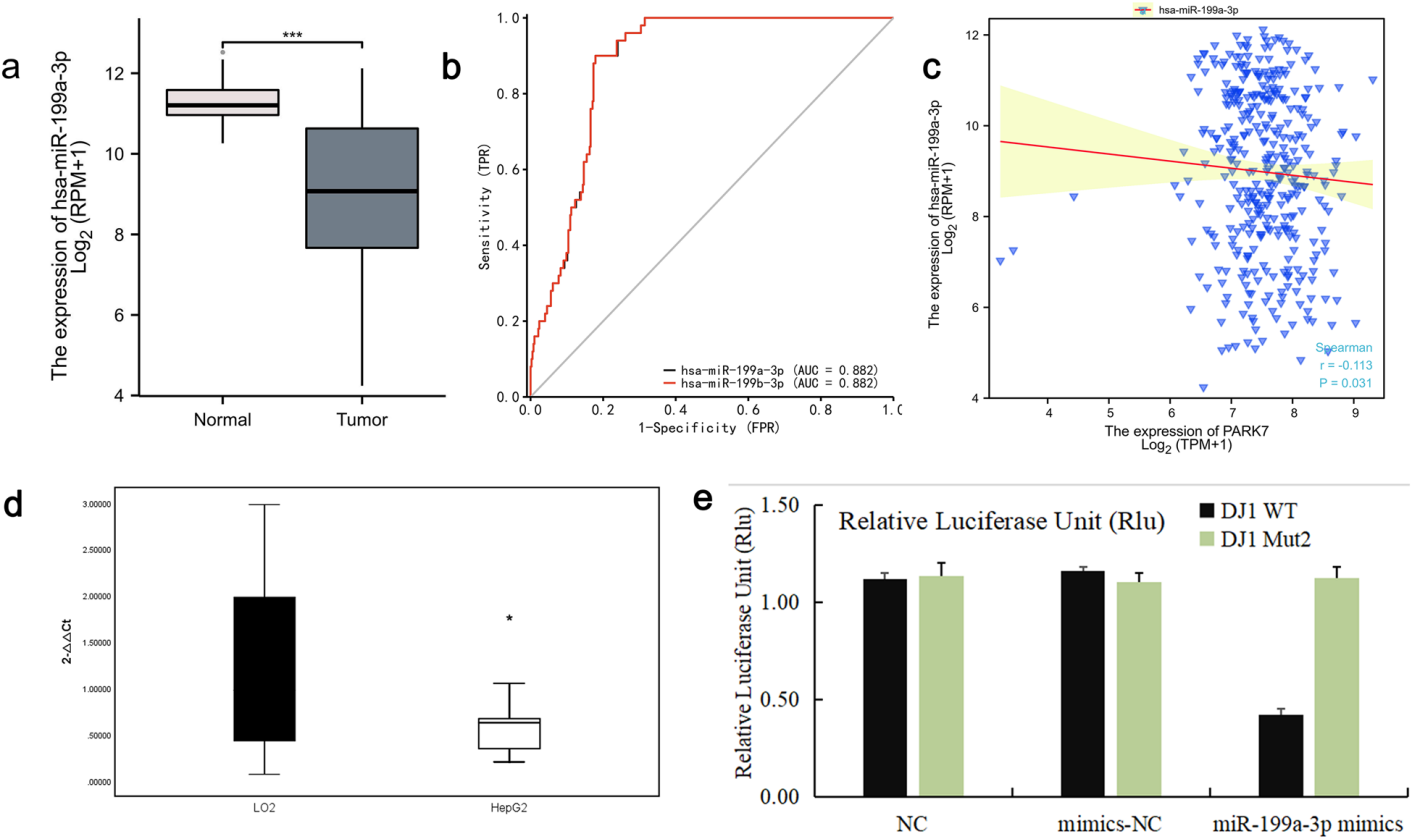
(**a**) miRNAs screening for Venn diagrams; (**b**) the expression of miR-199a-3p in TCGA-LIHC database; (**c**) the ROC curve of has-miR-199a/b-3p for HCC prediction value; (**d**) the molecular correlation analysis of has-miR-199a/b-3p and DJ-1; (**e**) the expression of miR-199a-3p in LO2 and HepG2 cell lines; (**f**) the targeted binding relationship between miR-199a-3p and DJ-1.

Noncoding RNA can affect disease progression by regulating gene expression, but there are few studies on the process of its regulation of DJ-1 gene expression affecting HCC patients. To shed further light on the regulatory mechanism of DJ-1 in HCC patients, we conducted miRWalk and TargetScan predictive analysis using the human gene DJ-1 as the target gene and Wayne diagram for miRNAs screening(Fig. 3a),We then analyzed the expression relationship of the overlapping miRNAs using TCGA-LIHC miRNA-seq and found that has-miR-199a/b-3p was poorly expressed in HCC tissues. Moreover, ROC curve analysis showed that has-miR-199a/b-3p had better diagnostic predictive value for HCC than other miRNAs, with an area under the curve of 88.2%, sensitivity of 90%, and specificity of 82.1% (Fig. 3b and c). Analysis of molecular correlation showed that hsa-miR-199a-3p exhibited a negative correlation with DJ-1, the Spearman correlation coefficients of r = −0.113 and *P* = 0.031 (Fig. 3d).Based on the bioinformation results presented above, we conducted Rt-PCR experiments on LO2 and HepG2 cell lines. Our findings indicate that has-miR-199a/b-3p was statistically significant in HepG2 (0.6432 ± 0.4388), which was lower than in LO2 (1.1979 ± 0.9177) (*P* < 0.05) (Fig. 3e). Bisluciferase gene report verification showed that the diluciferase activity of the wild-type DJ-1 gene plasmid vector and mutant DJ-1 gene plasmid vector in the hsa-miR-199a-3 pmimics group was significantly reduced compared with the diluciferase activity of the wild-type DJ-1 gene plasmid vector. This indicates that hsa-miR-199a-3p could bind to the wild-type DJ-1 gene and inhibit its expression, further supporting the existence of the targeted binding relationship mentioned above (Fig. 3f).

## Effect of DJ-1 and miR-199a-3p on HepG2 cell function

**Figure 4:**
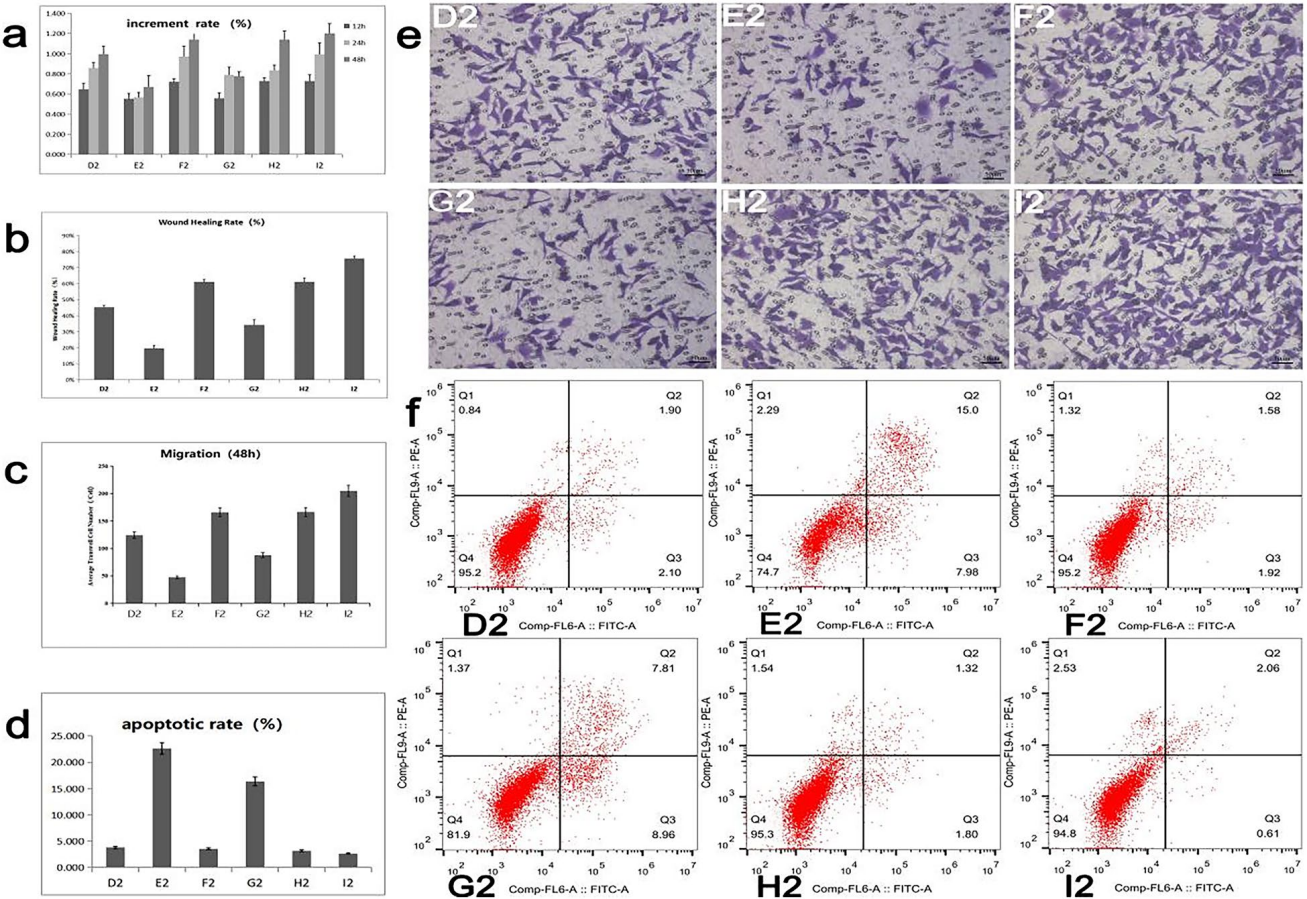
Proliferation, migration, invasion and apoptosis of HepG2 cells under different conditions, D2 blank control (HepG2), mimic group (E2), overexpression DJ-1 group (F2), mimic + overexpression DJ-1 group (G2), inhibitor group (H2), inhibitor + overexpression DJ-1 group (I2) The experiment was repeated three times, *P* < 0.05 was statistically significant, and the number of experiments *n* = 3.

The effects of DJ-1 gene, mi199a-3p and DJ-1 combined with miR199a-3p on HCC cell function were compared in HepG2 cell line. The detection methods and cell grouping were the same as 1.7. Cell function test results were shown in Fig. 4, The results showed that compared with the blank control group, overexpression of DJ-1 gene could promote the proliferation, migration and invasion of HepG2 cells and inhibit the process of apoptosis (as shown in groups 4a, b, c, d, e, f, D2 and F2). The intervention of miR-199a-3p mimics/inhibitors significantly inhibited the proliferation, migration and invasion of HepG2 cells. It promoted cell apoptosis (groups 4a,b,c,d,e,f, E2, H2), (groups 4a,b,c,d,e,f, G2, I2) and verified that miR199a-3p affected the cellular function of HepG2 cells by inhibiting the DJ-1 gene in both positive and negative aspects .

## Molecular verification of the expression relationship between hsa-miR-199a-3p and DJ-1 gene in HepG2 cells and signaling pathway

**Figure 5:**
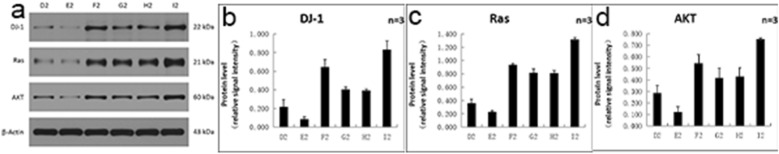
Shows the expression of DJ-1 gene, Ras protein and AKT protein in HepG2 cell lines before and after the regulation of miR-199a-3p by Westenblot technology, β-acting as the internal reference gene, the results are expressed in X- ± S, *P* < 0.05 is statistically significant, and the number of experimental replicates *n* = 3.

By reading the literature, we predict that Ras and PI3K/AKT may be the compensatory circuits of DJ-1 gene function during HCC progression. To verify this hypothesis, HepG2 cells were transfected with miR-199a-3p mimics, inhibitors and OE-DJ-1, and HepG2 cells were untransfected as the control group. The protein expressions of DJ-1, Ras, AKT and β-actin as internal reference genes in different groups were detected, and the cell group was the same as 1.7. The detection method is the same as 1.8. The expression analysis of DJ-1 gene in the six groups of cells (Fig. 5 a, b) showed that the expression of DJ-1 gene in the E2 group (0.087 ± 0.03) was significantly lower than that in the D2 group (0.219 ± 0.08), and the difference was statistically significant (*P* = 0.023, *P* < 0.05). The DJ-1 gene expression in the F2 (0.648 ± 0.08), G2 (0.407 ± 0.03), H2 (0.393 ± 0.02), and I2 (0.832 ± 0.1) groups was significantly increased compared with that in the D2 group, and the difference was statistically significant (*P* < 0.05). This indicates that miR-199a-3p mimic can significantly inhibit the expression of DJ-1 gene in HepG2 cells, while overexpression of DJ-1 gene can partially reverse miR-199a-3p, and miR-199a-3p inhibitor has the opposite effect.The expression of Ras protein molecules showed that the E2 group (0.231 ± 0.02) was lower than the D2 group (0.362 ± 0.06), and the difference was statistically significant (*P* = 0.002, *P* < 0.05). The expression of Ras protein in the F2 (0.938 ± 0.02), G2 (0.819 ± 0.06), H2 (0.81 ± 0.04), and I2 (1.317 ± 0.03) groups was significantly enhanced compared with that in the D2 group, and the difference was statistically significant (*P* < 0.05) (Fig. 5 a, c). This indicates that overexpression of DJ-1 gene can promote the expression of Ras, while miR-199a-3p can inhibit its expression.The expression of AKT signaling molecules showed that the E2 group (0.121 ± 0.05) was lower than the D2 group (0.286 ± 0.07), and the difference was statistically significant (*P* = 0.009, *P* < 0.05). The expression of AKT protein in the F2 (0.543 ± 0.08), G2 (0.419 ± 0.08), H2 (0.429 ± 0.08), and I2 (0.754 ± 0.01) groups was higher than that in the D2 group, and the difference was statistically significant (*P* < 0.05) (Fig. 5 a, d).The above results showed that DJ-1 gene overexpression could activate Ras and AKT protein, miR-199a-3p mimics could increase the expression of DJ-1 gene, and miR-199a-3p could inhibit the activation of oncogene Ras and AKT protein molecules by inhibiting the expression of DJ-1 gene.

## Discussion

Although the diagnosis and treatment of HCC has improved significantly, the five-year survival rate for HCC is still less than 15%^1–3^. The fundamental reason is that the molecular mechanism involved in the occurrence of HCC is extremely complex, and there is heterogeneity in HCC occurrence among patients, which may also be one of the reasons for the different treatment effect outcomes of patients. Therefore, comprehensively identifying the key target molecules involved in the progression of HCC, elucidating their pathogenesis and molecular mechanisms affecting drug sensitivity, and formulating precise individualized protocols according to patient heterogeneity may be effective methods to solve the poor prognosis and efficacy of HCC.

The DJ-1 (PARK7) gene is a mitogen-dependent oncogene present on the 36-site (1p36.12-1p36.33) of the short arm of human chromosome 1, encoding a highly conserved protein^8^, which is expressed in various tissue cells and is involved in different stages of cell growth and development (transcriptional regulation, cell transformation, oxidative stress response, chaperone, protease and mitochondrial regulation)^9–11^. The expression of DJ-1 gene can not only be used as a marker for mental cognitive diseases (such as Parkinson's disease, Alzheimer's disease^12,13^), but also participate in the occurrence and progression of various tumors (thyroid cancer^20^, ovarian cancer^41^, colon cancer^14,16,21^, liver cancer^25–31^, lung cancer^22,23^, renal clear cell carcinoma^42^, breast cancer^17^). Since Zhang D et al. first found that the expression of DJ-1 is associated with the occurrence of HCC associated with HBV infection^43^, the role of DJ-1 in the proliferation, invasion and metastasis of HCC^25–30^, has been paid more and more attention by researchers, and it is regarded as a potential oncogene therapy target for HCC^15^. In this study, we first analyzed the expression level of DJ-1 in TCGA database and clinical HCC tissue samples, and the results showed that the expression of DJ-1 in TCGA database and HCC tissue samples was significantly higher than that of normal tissues and paracancerous tissues, and was closely related to the clinical stage T (T1T2 vs T3T4), pathological grade (I II vs III IV.) and portal vascular invasion of HCC patients, which showed that the expression of DJ-1 could have an impact on HCC progression, and may be involved in HCC cell migration, The regulation of invasion is consistent with Wu F, Liu S et al.^25–27^, From the analysis of Biologics, we found that DJ-1 gene is related to early stage of patients, diagnosis prediction, vascular infiltration of blood vessels, indicating that it has a certain impact on HCC disease process and diagnosis, for the impact of DJ-1 gene on HCC development is not clear, in order to further explore and verify the impact of DJ-l gene on HCC, we used DJ-1 overexpression lentiviral vector to transfect HepG2 cells, the results showed that overexpression of DJ-1 genome can promote HepG2 cell proliferation, migration, The ability to invade and inhibit apoptosis, which indicates that the high expression of DJ-1 gene promotes the process of HCC cells, which is consistent with the conclusions of Wang Hui et al.^26,27^.

Kim MS et al^30^. found that DJ-1 can not only induce EMT transcription factor (TF) to activate the epithelial-mesenchymal transition (EMT) program through the TrkC/DJ-1/STAT3 signaling pathway, but also obtain anticancer drug resistance through TrkC-mediated inhibition of DJ-1 degradation^30^, and studies and other studies have shown that DJ-1 also receives the regulation of different miRNAs upstream in the form of target genes, which has an impact on tumor growth and drug sensitivity XL^31^. However, the mechanism by which the DJ-1 gene is regulated by upstream miRNA has not been reported.

miRNAs are a class of endogenous noncoding RNAs composed of 18–25 nucleotides, which can bind to the 3'-noncoding region (3'-UTR) of their specific target gene mRNA, and participate in the regulation of complex biological processes such as cell proliferation, differentiation, apoptosis, and carcinogenesis by regulating the expression of target genes^45–54^. In order to screen for possible upstream miRNAs involved in regulating DJ-1 expression, we found 4584 miRNAs targeting DJ-1 gene in the human genome through miRWalk predictive analysis. TargetScan predictive analysis found 157 miRNAs targeting the DJ-1 gene; Then, after superimposing the two databases by Wayne diagram, the HCC diagnostic ROC curve of the TCGA database was analyzed again to determine miR-199a/b-3p as a possible upstream miRNA of the DJ-1 gene for in-depth study.

A large number of studies have shown that miR-199a/b-3p is a tumor suppressor gene, which can inhibit the proliferation, invasion and drug resistance of tumor cells such as gastric cancer^32^, HCC^33–37^, breast cancer^38^, colorectal cancer^39^ and other tumor cells by regulating PAK4/MEK/ERK, PAK4/BCAR3 and other signaling pathways^55,56^^.^ Our analysis of TCGA data showed that the expression of miR-199a/b-3p in HCC tissue samples was significantly lower than that in adjacent tissues, and the results of HCC diagnostic ROC curve analysis also showed that miR-199a/b-3p had predictive value for HCC diagnosis. RT-qPCR experiments comparing normal human hepatocytes (LO2 cells) and human hepatoma cells (HepG2 cells) lines also showed that the expression of miR-199a/b-3p in HepG2 cell lines was significantly down-regulated, and these results showed that miR-199a/b-3p expression was low and HCC, consistent with the conclusions of Li, Zhenyang et al. ^33–36^. At the same time, our analysis of the molecular correlation results of TCGA database showed that miR-199a/b-3p and DJ-1 gene had a negative correlation in HCC, which may be regulated by targeting DJ-1, and its role and potential mechanism of targeting DJ-1 in HCC have not been reported. In this study, the interaction between miR-199a/b-3p and DJ-1 gene was first verified by diluciferase gene report, and the results showed that miR-199a/b-3p could target binding to wild-type DJ-1 gene and significantly downregulate DJ-1 gene expression, confirming the targeting relationship between miR-199a/b-3p and DJ-1 gene. We also found that miR-199a/b-3p mimics significantly reduced DJ-1 expression in HepG2 cell lines, in order to further verify the targeting effect of miR-199a/b-3p on the DJ-1 gene, We intervened miR-199a/b-3pmimc and miR-199a/b-3pinhibitor into HepG2 cells and transfected HepG2 cells overexpressing DJ-1 plasmids, respectively, and found that miR-199a-3pmimc can inhibit the proliferation, migration and invasion ability of HepG2 cells by inhibiting DJ-1 gene expression and promote the apoptosis process (*P* < 0.005).This finding reconfirmed the negative regulatory relationship between miR-199a/b-3p and DJ-1 gene, and had an impact on the progression of HCC.

Numerous studies have shown that DJ-1 is involved in regulating HCC proliferation, apoptosis, and invasion transfer mainly by influencing PTEN, MAPK, and AKT signaling pathways^21,27,28,30,57^, and the components of these signaling cascades have been used as the main targets for therapeutic intervention. Guo XL et al. have shown that DJ-1 regulates PI3K/AKT signaling pathway is closely related to tumor apoptosis and invasion^31^, while oncogene Ras protein can synergize with DJ-1 gene and act together on PI3K/AKT pathway, and the role of activated Ras, AKT on the imbalance of PI3K/AKT signaling pathway in the pathogenesis of various tumors and the components of its signaling cascade have been gradually demonstrated as therapeutic targets for liver cancer. So is there a miR-199a/b-3p targeting DJ-1 gene and Ras protein to regulate HCC cell proliferation, apoptosis, and migration invasion by inhibiting the PI3K/AKT signaling pathway? We further found through WB experiments that overexpression of DJ-1 gene can enhance the expression of oncogene Ras and PI3K/AKT signaling pathway key protein molecule AKT, while miR-199a/b-3pmimc can downregulate the expression of oncogene Ras protein molecule and inhibit the activation of PI3K/AKT signaling pathway key protein AKT. Conversely, increasing the expression of DJ-1 can reverse the inhibitory effect of miR-199a/b-3pminic on HepG2 cell function.

In summary, these results show that miR-199a/b-3p can bind to DJ-1 gene targeting and inhibit the activation of PI3k/AKT signaling pathway by downregulating the expression of Ras protein, inhibit HepG2 cell proliferation, proliferation and migration invasion, and promote HepG2 cell apoptosis, which verifies our experimental hypothesis. To our knowledge, this is the first demonstration of the miR-199a/b-3p/DJ-1/Ras/PI3k/AKT axis as a study involved in regulating HepG2 cell proliferation, apoptosis, and migration invasion. In addition, our findings are significant because it provides a better understanding of the pathogenesis of HCC and provides a new approach to predicting and assessing prognosis and potential therapeutic targets for HCC.

## Conclusions

In summary, our study suggests that miR-199a/b-3p, as an upstream regulatory molecule of DJ-1, may inhibit cell proliferation and invasion of HCC and promote cell apoptosis through a novel compensatory signaling pathway involving miR-199a/b-3p\DJ-1\Ras\PI3K/AKT.

### Supplementary Information

Supplementary Information 1.

## Data Availability

The datasets used during the current study are available from the corresponding author on reasonable request.
